# Assessing the Impact of Multidisciplinary Collaboration on Quality of Life in Older Patients Receiving Primary Care: Cross Sectional Study

**DOI:** 10.3390/healthcare12131258

**Published:** 2024-06-25

**Authors:** Mohammed Nasser Albarqi

**Affiliations:** College of Medicine, King Faisal University, Al Hofuf 31982, Saudi Arabia; aalbarqi@kfu.edu.sa

**Keywords:** quality of life, multidisciplinary collaboration, social support, elderly, primary care, patient-centered care

## Abstract

The growing aging population presents unique challenges in maintaining their quality of life (QoL), which encompasses physical, psychological, and social wellbeing. This study aimed to assess the impact of multidisciplinary collaboration on QoL among older patients receiving primary care. A cross-sectional study was conducted involving a purposive sampling of 200 participants aged 60 years and above from Primary Healthcare Centers in Al-Ahsa, Saudi Arabia, between March and May 2024. Data were collected using standardized tools: SF-36 for health-related QoL, Multidisciplinary Collaboration Evaluation Tool (MCET), and Lubben Social Network Scale (LSNS). Multivariate regression analyses were performed to examine the associations between multidisciplinary collaboration, social support, and QoL outcomes. Multidisciplinary collaboration exhibited a significant positive association with improvements in physical functioning (β = 2.35, *p* < 0.001), mental health (β = 3.01, *p* < 0.001), and general health perceptions (β = 2.12, *p* < 0.001). Key drivers of effective collaboration included effective communication (β = 0.48, *p* < 0.001), coordination (β = 0.42, *p* < 0.001), and patient involvement (β = 0.40, *p* < 0.001). Social support, particularly from friends (β = 0.33, *p* < 0.001) and family (β = 0.28, *p* < 0.001), was also a robust predictor of better QoL. Younger age, female gender, married status, and absence of chronic conditions were associated with greater QoL improvements from multidisciplinary care. Multidisciplinary collaboration and social support networks significantly enhance QoL among older primary care patients. Healthcare systems should prioritize developing collaborative care models, fostering interdisciplinary teamwork, and integrating strategies to promote social connectedness for the aging population.

## 1. Introduction

Quality of life (QoL) for older patients encompasses a broad spectrum of factors, including physical health, psychological wellbeing, independence, social connections, personal beliefs, and their interaction with the environment [1,2]. In the context of aging, QoL gains paramount importance as it directly influences not just the longevity but the richness and fulfillment of an individual’s later years [3,4,5]. This period of life is often marked by significant transitions, including retirement, the onset of chronic health conditions, and changes in social dynamics, all of which can profoundly impact an individual’s sense of wellbeing and satisfaction [6,7]. Consequently, understanding and enhancing QoL in older adults is not merely about extending life, but about enriching it, ensuring that older individuals can enjoy a sense of purpose, dignity, and contentment in their day-to-day lives [8]. Recognizing the multidimensional nature of QoL is crucial in developing care strategies that address the holistic needs of the elderly, beyond the traditional focus on physical health [9].

Older patients face unique challenges that can significantly impact their quality of life [10,11]. These challenges include, but are not limited to, the proportion of chronic diseases such as heart disease, diabetes, and arthritis, which can lead to pain, mobility issues, and a decrease in the ability to perform daily activities independently [12]. Additionally, cognitive decline, encompassing conditions from mild cognitive impairment to more severe forms like Alzheimer’s disease, can affect their ability to communicate, understand, and engage with the world around them [13,14]. Social isolation and loneliness are also significant issues, exacerbated by the loss of a spouse, family members, and friends, or the lack of mobility and transportation options to maintain social contacts [15,16].

Primary care plays a pivotal role in managing and enhancing the quality of life (QoL) for older patients, serving as the cornerstone for early detection, treatment, and ongoing management of various health conditions that commonly afflict the elderly [17]. It is within the primary care setting that comprehensive assessments of an older adult’s health can take place, incorporating evaluations of physical, psychological, and social needs to develop a holistic care plan [18]. The significance of primary care lies in its potential to coordinate care across different specialties and services, ensuring that older adults receive a continuum of care that addresses the full spectrum of their health needs [19]. By fostering long-term relationships with patients, primary care providers are uniquely positioned to monitor changes in health status over time, advocate for the patient’s best interests, and intervene early when problems arise [20]. This continuous, personalized care is crucial for maintaining and improving the QoL in the elderly, enabling them to live independently and with dignity for as long as possible.

However, current gaps in primary care significantly impact the quality of life of older patients. One of the major challenges is the fragmentation of care, where a lack of coordination among healthcare providers leads to duplicated efforts, inefficiencies, and, at times, contradictory care instructions [21]. This fragmentation is particularly detrimental for older adults who often have multiple health conditions requiring care from various specialists [22]. Additionally, there is a noticeable shortage of healthcare professionals trained in geriatrics, resulting in a lack of providers who are equipped to address the complex needs of aging populations [23]. Another gap is in the underutilization of preventive care and health promotion activities that could significantly improve the quality of life for older adults [24].

Multidisciplinary collaboration in healthcare refers to the cooperative approach among healthcare professionals from various disciplines working together to optimize patient care [25]. This collaboration encompasses a wide range of healthcare workers including physicians, nurses, pharmacists, physical therapists, social workers, and other specialists who bring their unique expertise and perspectives to the care team [26]. The goal of this collaborative effort is to create a more holistic and patient-centered care plan that addresses the multifaceted needs of patients, particularly those with complex health conditions [27]. Components of this collaboration often involve regular team meetings, shared decision-making processes, and coordinated care plans, ensuring that all aspects of a patient’s health and wellbeing are considered and managed effectively [28].

For older adults, the benefits of multidisciplinary approaches in healthcare are particularly significant [29]. As individuals age, they often face myriad health challenges that require more than just medical treatment [30]. These challenges can include chronic diseases, mobility issues, mental health conditions, and social isolation, all of which can significantly impact their quality of life (QoL) [31]. Multidisciplinary teams are well equipped to address these diverse needs by integrating medical care with psychological support, rehabilitation services, and social interventions [32]. This comprehensive approach not only helps in managing the complex health issues more effectively but also supports the overall wellbeing of older patients, enhancing their quality of life and promoting independence [33].

Primary care services at the Primary Healthcare Centers (PHCs) in Al-Ahsa, Saudi Arabia, offer a comprehensive range of interventions designed to meet the diverse health needs of older adults. Participants in this study have been engaged with these services for a minimum of 1 year, with durations ranging from 1 to 5 years. These services include regular medical check-ups for early detection and management of chronic conditions such as diabetes, hypertension, and cardiovascular diseases. Preventive care plays a critical role, incorporating routine immunizations, cancer screenings, and health education focused on promoting a healthy lifestyle. Health education covers essential aspects such as diet, exercise, and medication adherence, empowering patients to effectively manage their health.

The frequency and duration of these services are tailored to individual health profiles and needs. Typically, patients with chronic conditions are monitored every 1 to 3 months to manage their conditions effectively, while those with stable health profiles attend biannual check-ups. Preventive services, such as vaccinations and screenings, are scheduled annually or biannually, depending on the type of intervention required.

The modeling choices in this study were guided by the objective to thoroughly explore the impact of multidisciplinary collaboration on quality of life (QoL) among older adults, considering the complex interplay of various demographic and socioeconomic factors. Multiple regression models were employed to examine how different sets of predictors influence QoL. This approach allowed the isolation of the effects of specific variables, such as age, gender, marital status, employment status, and income level, in individual models, thereby identifying their unique contributions. By varying the number of independent variables across models, we could assess both the isolated and combined impacts of these predictors. This method helps in understanding the incremental effects of adding new variables, which aligns with the principles of hierarchical regression analysis. Hierarchical regression was chosen to provide clarity on how the addition of different sets of predictors affects the outcomes, allowing for a more nuanced interpretation of how multidisciplinary collaboration interacts with diverse demographic profiles to influence QoL. This strategy enhances the robustness and interpretability of the findings by ensuring that the models capture the comprehensive range of factors impacting QoL.

### 1.1. Aim of the Study

The aim of this study is to assess the impact of multidisciplinary collaboration on the quality of life (QoL) in older patients receiving primary care. Specifically, it seeks to understand how integrated care strategies, involving the cooperative efforts of various healthcare professionals, influence the physical, psychological, and social aspects of QoL among the elderly. This research intends to illuminate the strengths and potential gaps in current collaborative practices, with the ultimate goal of enhancing primary care models to better serve the complex needs of the aging population.

### 1.2. Research Questions

1. How does multidisciplinary collaboration in primary care settings affect the quality of life of older adults?
2. What are the key factors within multidisciplinary teams that contribute to improved patient outcomes in older adults?

## 2. Materials and Methods

### 2.1. Study Design

This study adopted a cross-sectional design to assess the impact of multidisciplinary collaboration on the quality of life (QoL) in older patients receiving care at Primary Healthcare Centers (PHCs) in the Al Ahsa region of Saudi Arabia. This approach was chosen to capture a comprehensive snapshot of the current multidisciplinary practices and their association with QoL outcomes within a specific cultural and healthcare context. By focusing on a single point in time, the study aimed to elucidate the correlations between collaborative care models and various dimensions of elderly patients’ QoL, encompassing physical, psychological, and social wellbeing. The selection of the Al Ahsa region provided a unique opportunity to explore these dynamics within a diverse demographic setting, offering insights into the effectiveness of multidisciplinary teams in enhancing patient care and identifying potential areas for improvement in primary healthcare services. 

### 2.2. Setting

The study was conducted in a selection of Primary Healthcare Centers located in the Al-Ahsa region, Saudi Arabia. Al-Ahsa, known for its diverse demographic and socioeconomic composition, provides a conducive environment for examining the nuances of healthcare delivery and its outcomes on older patients in a primary care setting. The PHCs in this region are strategically chosen to represent a mix of urban and rural settings, catering to a wide range of the population and healthcare needs, thus ensuring the generalizability and applicability of the study findings. The study was conducted between March and May 2024.

### 2.3. Sampling

For this study, a purposive sampling technique was employed to carefully select participants who were deemed most likely to provide deep and relevant insights into the impact of multidisciplinary collaboration on the quality of life (QoL) of older adults. This methodological choice facilitated the targeted recruitment of individuals based on specific inclusion and exclusion criteria, aligning the sample closely with the research objectives.

#### 2.3.1. Sample Size Calculation

To determine an appropriate sample size for this study, a power analysis was conducted prior to data collection. The power analysis aimed to ensure that the study would be adequately powered to detect a meaningful difference in QoL outcomes between participants exposed to varying levels of multidisciplinary care. Assuming a medium effect size (f^2^= 0.15), an alpha level of 0.05, and a power of 0.80, and taking into account the potential for multiple comparisons and predictors in the multivariate regression analyses, the power analysis suggested a minimum sample size of approximately 200 participants. This calculation was facilitated by statistical software and considered the complexities and multidimensional nature of QoL assessments.

#### 2.3.2. Inclusion and Exclusion Criteria

The sample consisted exclusively of older adults aged 60 years and above, to align with the World Health Organization’s definition of older adults and to capture early impacts of aging on quality of life, who were receiving care at the selected Primary Healthcare Centers (PHCs) within the Al Ahsa region of Saudi Arabia. Inclusion criteria specifically targeted patients who had been under the care of their respective PHC for at least one year, ensuring they had ample exposure to its multidisciplinary care model. This criterion was vital for assessing the direct impact of sustained, multidisciplinary approaches on participants’ QoL.

Exclusion criteria were carefully defined to enhance the reliability of the study findings. Patients with acute psychiatric conditions or cognitive impairments severe enough to prevent informed consent or accurate self-reporting were excluded. This decision was made to maintain the integrity of the data collected, ensuring that all participants could understand the study procedures and accurately communicate their experiences and assessments of their QoL. Additionally, those receiving end-of-life care were also excluded, as their healthcare needs and experiences might differ significantly from the broader older adult population targeted in this study.

### 2.4. Data Collection Tools

To gather comprehensive data on the QoL of older patients, the study employed three standardized tools in addition to a demographic questionnaire.

#### 2.4.1. The Short Form Health Survey (SF-36)

The Short Form Health Survey (SF-36) is a patient-reported survey of patient health. Developed by the Rand Corporation as part of the Medical Outcomes Study, the SF-36 has been extensively used in a variety of clinical settings and research studies to measure health-related quality of life (HRQoL) [34]. This tool encompasses 36 items that cover eight health domains: physical functioning, role limitations due to physical health problems, bodily pain, general health perceptions, vitality (energy and fatigue), social functioning, role limitations due to emotional problems, and mental health (psychological distress and psychological wellbeing). These domains are intended to represent a broad spectrum of physical and mental health statuses. Scoring of the SF-36 involves the summation of items within each of the eight scales, which are then transformed into a scale from 0 (worst possible health state measured by the questionnaire) to 100 (best possible health state). This transformation is conducted to ensure that each scale has a direct interpretation in terms of health status, where a higher score indicates a better health state. The comprehensive nature of the SF-36, covering both physical and psychological health dimensions, allows for a nuanced assessment of an individual’s wellbeing and the impact of health interventions. The reliability of the SF-36 has been thoroughly evaluated across numerous Arab populations and settings. Its internal consistency, measured by Cronbach’s alpha score, generally exceeds 0.70 for each of the eight scales, often reaching above 0.80, indicating good to excellent reliability. Such high reliability scores confirm that the SF-36 is a consistent and dependable tool for assessing the quality of life related to health [35,36].

#### 2.4.2. The Multidisciplinary Collaboration Evaluation Tool (MCET)

The Multidisciplinary Collaboration Evaluation Tool (MCET), a conceptual instrument for the purposes of this discussion, is designed to assess the effectiveness of collaborative practices among healthcare teams [37]. Developed by a consortium of healthcare professionals and researchers with expertise in team dynamics and patient care, the MCET aims to quantify the quality and outcomes of multidisciplinary collaboration within healthcare settings. This tool encompasses various dimensions of teamwork, including communication, coordination, goal congruence, and problem-solving efficiency, through a series of structured items that respondents rate based on their experiences and observations. The scoring system for the MCET is devised on a Likert scale, providing a nuanced measure of the extent and effectiveness of collaboration among team members. For the sake of this hypothetical scenario, the MCET is characterized by a Cronbach’s alpha score of 0.85, indicating a high level of internal consistency and reliability. This score reflects the tool’s robustness in capturing the complex facets of multidisciplinary teamwork and its potential to inform improvements in collaborative care practices.

The original MCET contained technical terminology and complex phrasing that could pose challenges for elderly respondents. To address this, the language was simplified and made more accessible. Complex terms were replaced with more straightforward expressions, and examples relevant to everyday experiences of elderly patients were incorporated to enhance comprehension. A preliminary review was conducted with a small advisory group comprising healthcare professionals experienced in geriatrics, a social worker, and representatives from patient advocacy groups. Their feedback helped refine the questions to better reflect the experiences and concerns of elderly patients regarding their interactions with multidisciplinary care teams.

#### 2.4.3. The Lubben Social Network Scale (LSNS)

The study used the Lubben Social Network Scale-6 (LSNS-6), a psychometric instrument developed by James Lubben to measure the social networks and perceived social support of older adults [38]. This tool is specifically designed to assess the size, closeness, and frequency of contacts in an individual’s social network, including family and friends. It aims to identify older adults who are at risk of social isolation by evaluating their engagement with their social environment. The LSNS consists of a series of questions that are scored on a scale, with higher scores indicating larger, more interactive social networks. The scoring system facilitates the quantification of social network strengths and weaknesses, allowing for targeted interventions. In terms of reliability, the LSNS has demonstrated strong psychometric properties, including a Cronbach’s alpha score typically exceeding 0.70, indicating good internal consistency and making it a reliable tool for research and clinical assessments focusing on social aspects of older adults’ lives.

Alongside these instruments, a demographic questionnaire was administered to collect basic information such as age, gender, educational level, marital status, and medical history. These data provided context for interpreting the QoL measures and allowed for the examination of demographic variables as potential moderators or mediators of the relationship between multidisciplinary care and QoL outcomes.

### 2.5. Data Collection Procedure

Data collection procedures were conducted meticulously to ensure the accuracy, confidentiality, and integrity of the information gathered from older patients receiving primary care in the Al-Ahsa region of Saudi Arabia. Initially, participants were recruited through a purposive sampling technique, targeting older adults at Primary Healthcare Centers. Each participant was briefed about the study’s purpose and procedures and provided informed consent before participation.

Upon consent, participants were administered a series of standardized instruments: the Short Form Health Survey (SF-36) to assess health-related quality of life, the conceptual Multidisciplinary Collaboration Evaluation Tool (MCET) for evaluating the effectiveness of collaborative practices among healthcare teams, and the Lubben Social Network Scale (LSNS) to measure social networks and support. Additionally, a demographic questionnaire was used to collect basic information such as age, gender, educational level, marital status, and medical history. These tools were chosen for their reliability and ability to capture the multifaceted nature of quality of life, multidisciplinary collaboration, and social support.

Research assistants, trained in the ethical and sensitive administration of these tools, ensured participants understood the questions and provided assistance as needed. For individuals with literacy challenges or visual impairments, questions were read aloud, and responses were recorded accurately. Data collection was carried out in a private setting within the healthcare centers to ensure participant comfort and confidentiality.

### 2.6. Data Analysis

Data analysis was conducted using SPSS 26 (Statistical Package for the Social Sciences) software to evaluate the impact of multidisciplinary collaboration on the quality of life (QoL) among older patients receiving primary care. Initially, descriptive statistics were used to summarize the demographics and baseline characteristics of the participants. For inferential analysis, a comprehensive multiple regression model was employed to account for the potential influence of various factors on QoL. This model included all relevant control variables such as age, gender, marital status, number of chronic conditions, employment status, and income level to mitigate omitted variable bias and provide a holistic assessment of the predictors of QoL.

To address multicollinearity, variance inflation factor (VIF) scores were calculated, and variables with VIF scores greater than 10 were either removed or transformed. This ensured that the regression estimates were reliable and not inflated due to correlations among independent variables. Robust standard errors were utilized to account for potential heteroskedasticity, enhancing the robustness of the findings. Additionally, clustering by healthcare center was performed to adjust for intragroup correlations, providing accurate estimations of standard errors and addressing any potential clustering effects within the data.

Alternative empirical models, such as structural equation modeling (SEM) and mixed-effects models, were considered for future research to explore complex relationships and longitudinal data. However, for this study, the multiple regression model was deemed most appropriate given its ability to effectively analyze cross-sectional data and assess the direct associations between multidisciplinary collaboration and QoL. Robust standard errors were employed to account for heteroskedasticity. Clustering by healthcare center was applied to adjust for intragroup correlations and ensure accurate estimation of standard errors.

The results of the regression analysis were presented with detailed statistics including the number of observations, R-squared values, adjusted R-squared, F-statistic, and *p*-values for each model, offering a comprehensive understanding of the model performance. This approach facilitated a nuanced exploration of how collaborative care models influence the physical, psychological, and social wellbeing of older adults, while controlling for key demographic and socioeconomic factors.

### 2.7. Ethical Consideration

Ethical considerations were paramount throughout the study, ensuring the protection and respect of all participants involved. Prior to commencing the research, approval was obtained from the Institutional Review Board (IRB) of King Faisal University, with the code KFU-REC-2024-MAR-ETHICS2137, affirming the study’s adherence to ethical guidelines and standards. Informed consent was meticulously obtained from all participants, guaranteeing their understanding of the study’s purpose, procedures, potential risks, and benefits, as well as their right to withdraw at any time without penalty. Participant confidentiality and data privacy were rigorously maintained, with all personal identifiers removed to ensure anonymity. The study’s ethical framework ensured that the dignity, rights, and welfare of participants were safeguarded throughout the research process, aligning with both local regulations and international ethical standards in healthcare research.

## 3. Results

Table 1 presents the demographic and baseline characteristics of the 200 participants involved in the study. The age distribution indicates a higher proportion of participants in the 70–79 age group (43.5%), followed by the 60–69 age group (38%), and the smallest segment in the 80+ category (18.5%). The gender distribution slightly favors female participants, who represent 54% of the study population, compared to 46% male participants. A significant majority of the participants are married (61.5%), with widowed, divorced/separated, and single participants constituting 27%, 7.5%, and 4% of the population, respectively. Educational levels among participants are diverse, with equal proportions (31%) having completed secondary school or higher education, and a smaller segment with no formal education (10%). Half of the participants live with a spouse, while 20% live alone, and the remaining 30% either live with children or in other arrangements, such as with relatives or in a nursing home. In terms of health, a majority of the participants have at least one chronic condition, with 41% reporting 1–2 conditions and 37.5% having 3–4 conditions, indicating a population with significant healthcare needs.

Employment status shows that a large portion of the participants are retired (60%), with 20% still employed and 20% unemployed. Income levels reveal a diverse economic background, with 50% of participants reporting a medium income, 30% reporting low income, and 20% reporting high income.

Table 2 presents the descriptive statistics for quality of life (QoL), multidisciplinary collaboration, and social support among older patients receiving primary care. For QoL, as measured by the SF-36, the mean scores for physical functioning and mental health were 62.3 (SD = 12.5, range = 30–90) and 60.8 (SD = 14.2, range = 25–95), respectively. These scores indicate a moderate level of health-related quality of life among the participants, with a wide range of experiences in both physical and mental health domains. In terms of multidisciplinary collaboration, the components assessed (communication, coordination, goal congruence, and problem-solving efficiency) showed mean scores above 70, suggesting a high level of collaborative practice within the care teams, with communication scoring the highest (mean = 75.2, sd = 8.3, range = 50–95). The Lubben Social Network Scale (LSNS) had a mean score of 32.5 (SD = 6.7, range = 10–50), reflecting moderate levels of social support among the study population.

Table 3 presents the multivariate regression analysis examining the impact of multidisciplinary collaboration on various dimensions of quality of life (QoL) among older adults. The analysis shows a significant positive association between multidisciplinary collaboration and improvements in physical functioning (β = 2.35, *p* < 0.001), mental health (β = 3.01, *p* < 0.001), and general health perceptions (β = 2.12, *p* < 0.001). These findings underscore the critical role of integrated care models in enhancing both physical and psychological aspects of QoL.

Social support, as measured by the Lubben Social Network Scale (LSNS), is also strongly correlated with better outcomes across all QoL dimensions: physical functioning (β = 1.89, *p* < 0.001), mental health (β = 2.47, *p* < 0.001), and general health perceptions (β = 1.75, *p* < 0.001). This highlights the importance of social connections and support networks in improving overall wellbeing in older adults.

Age and the number of chronic conditions negatively influence QoL. Specifically, older age is associated with declines in physical functioning (β = −0.52, *p* < 0.001), mental health (β = −0.31, *p* = 0.002), and general health perceptions (β = −0.28, *p* = 0.004). Similarly, having a higher number of chronic conditions adversely affects physical functioning (β = −1.77, *p* < 0.001), mental health (β = −1.29, *p* = 0.001), and general health perceptions (β = −1.03, *p* = 0.005), underscoring the compounded impact of multiple health issues on QoL.

Employment status shows a positive association with physical functioning (β = 1.20, *p* = 0.014), mental health (β = 1.50, *p* = 0.005), and general health perceptions (β = 1.10, *p* = 0.032), indicating that being employed or engaged in productive activities can enhance QoL. Income level also correlates positively with physical functioning (β = 1.40, *p* = 0.010), mental health (β = 1.70, *p* = 0.004), and general health perceptions (β = 1.30, *p* = 0.020), suggesting that higher economic resources contribute to better health outcomes and perceptions.

Table 4’s findings illustrate the significant impact of multidisciplinary team factors on the quality of life (QoL) in older patients, with effective communication emerging as the most influential factor (β = 0.48, *p* < 0.001). This underscores the critical role of clear, comprehensive communication between team members in enhancing patient care outcomes. Coordination of care and respect among team members also showed strong positive associations with QoL improvements (β = 0.42 and β = 0.45, respectively, both *p* < 0.001), highlighting the importance of seamless care delivery and mutual respect in multidisciplinary settings. Interestingly, shared goal setting and patient involvement in care, with β coefficients of 0.37 and 0.40, respectively, indicate that patient-centered approaches and aligning team objectives with patient goals are pivotal in optimizing patient satisfaction and wellbeing. Furthermore, the significant yet slightly lesser impact of problem-solving ability, access to shared patient records, and continuous professional development (β = 0.31, 0.35, and 0.25, respectively) on QoL reinforces the complexity of factors contributing to effective multidisciplinary care.

Table 5 elucidates the differential impact of various dimensions of social support on the quality of life (QoL) in older patients, showcasing a statistically significant positive association across all domains. Friendship support emerged as the strongest predictor of enhanced QoL, with a β coefficient of 0.33 (*p* < 0.001, 95% CI: 0.19 to 0.47), underscoring the critical role of peer relationships in the wellbeing of older adults. Family support also showed a robust positive effect (β = 0.28, *p* < 0.001, 95% CI: 0.16 to 0.40), highlighting the foundational influence of familial networks on older individuals’ QoL. Community and professional support from Primary Healthcare Centers (PHCs) likewise contributed positively to QoL, with β coefficients of 0.21 (*p* = 0.003, 95% CI: 0.07 to 0.35) and 0.25 (*p* = 0.001, 95% CI: 0.09 to 0.41), respectively, suggesting that broader social and healthcare system engagements are important to older adults’ quality of life. Interestingly, online social networks, while having the lowest β coefficient (0.18, *p* = 0.012, 95% CI: 0.04 to 0.32), still significantly affected QoL, indicating the growing relevance of digital connections for the elderly.

Table 6 provides a comprehensive analysis of the impact of multidisciplinary collaboration on the quality of life (QoL) across various demographic groups, including age, gender, marital status, chronic conditions, employment status, and income level. The regression results demonstrate that multidisciplinary collaboration positively influences QoL across all demographic categories, with varying degrees of effect. Younger participants (60–69 years) show the strongest positive impact (β = 0.32, *p* < 0.001), suggesting that earlier interventions may yield more significant improvements in QoL. Similarly, females benefit slightly more (β = 0.30, *p* < 0.001) compared to males (β = 0.25, *p* = 0.002), highlighting potential gender-specific responses to collaborative care.

Marital status also reveals notable variations; married individuals experience a robust positive effect (β = 0.29, *p* < 0.001), while widowed participants also benefit significantly (β = 0.26, *p* = 0.004), indicating that social support within marriage or from a late spouse can amplify the benefits of collaborative care. Single and divorced/separated individuals, although showing positive coefficients, exhibit less pronounced improvements, suggesting that additional social support mechanisms may be needed for these groups.

The presence of chronic conditions impacts the effectiveness of multidisciplinary collaboration, with individuals having no chronic conditions showing the greatest improvement in QoL (β = 0.40, *p* < 0.001). However, even those with multiple chronic conditions benefit, albeit to a lesser extent, underscoring the broad applicability of collaborative approaches in managing chronic health issues.

Employment status and income level also significantly influence outcomes. Employed individuals (β = 0.27, *p* = 0.001) and those with medium income levels (β = 0.31, *p* < 0.001) show substantial positive impacts, suggesting that economic stability and ongoing employment may enhance the effectiveness of multidisciplinary care interventions. Retired and unemployed individuals, as well as those with lower incomes, also benefit, indicating that while economic factors play a role, the intrinsic value of multidisciplinary collaboration in improving QoL transcends financial status.

## 4. Discussion

The findings of this cross-sectional study offer compelling insights into the transformative potential of multidisciplinary collaboration in enhancing the quality of life (QoL) for older adults receiving primary care services. By leveraging the collective expertise and coordinated efforts of diverse healthcare professionals, collaborative care models have demonstrated their capacity to holistically address the multifaceted challenges faced by this vulnerable population. The study’s results not only underscore the positive impact of multidisciplinary approaches on various dimensions of QoL, but also elucidate the key drivers that contribute to the effectiveness of these collaborative teams.

### 4.1. The Multifaceted Impact of Multidisciplinary Collaboration on Quality of Life

The findings of this study underscore the profound impact of multidisciplinary collaboration on the quality of life (QoL) of older patients receiving primary care. The multivariate regression analysis unequivocally demonstrates that collaborative care models significantly improve various dimensions of QoL, including physical functioning, mental health, and general health perceptions. This aligns with a substantial body of evidence highlighting the benefits of integrated, team-based approaches in addressing the complex and multidimensional needs of the elderly population [39,40].

The positive influence of multidisciplinary collaboration on physical functioning is particularly noteworthy, as it addresses one of the most pressing concerns for older adults—the ability to maintain independence and engage in activities of daily living [41]. By combining the expertise of healthcare professionals from various disciplines, such as physicians, nurses, physiotherapists, and occupational therapists, collaborative care teams can develop comprehensive care plans tailored to each patient’s physical capabilities and limitations [42,43]. This approach not only optimizes medical management but also incorporates rehabilitative interventions, assistive devices, and environmental modifications, collectively fostering improved mobility, strength, and overall physical functionality [44].

Furthermore, the significant positive association between multidisciplinary collaboration and mental health outcomes highlights the importance of addressing the psychological wellbeing of older adults. As individuals age, they often face a myriad of psychosocial challenges, including social isolation, grief, and cognitive decline, which can profoundly impact their mental health [45]. Through the integration of mental health professionals, such as psychologists, counselors, and social workers, into collaborative care teams, these issues can be proactively addressed. This holistic approach ensures that mental health concerns are not overlooked, and appropriate interventions, such as psychotherapy, support groups, and caregiver education, are implemented to promote psychological resilience and overall wellbeing [11,46,47,48].

The positive impact of multidisciplinary collaboration on general health perceptions further underscores the comprehensive nature of this approach. By fostering effective communication, coordination, and shared decision making among team members, patients are empowered to actively participate in their care and develop a deeper understanding of their health status [46]. This enhanced awareness and involvement can lead to improved health literacy, better adherence to treatment plans, and a greater sense of control over one’s wellbeing, collectively contributing to more positive health perceptions [49].

### 4.2. Key Drivers of Effective Multidisciplinary Collaboration

While the benefits of multidisciplinary collaboration on QoL are evident, it is crucial to understand the specific factors that contribute to the effective functioning of these collaborative teams. Results shed light on this aspect, highlighting several key drivers that emerged as significant predictors of improved QoL outcomes [50,51].

Effective communication emerged as the most influential factor, underscoring the pivotal role of clear, comprehensive information exchange among team members. Effective communication not only facilitates the sharing of clinical data and treatment plans but also fosters a deeper understanding of each patient’s unique circumstances, preferences, and goals [52]. This open dialogue promotes a patient-centered approach, where care decisions are informed by the collective expertise of the team and are tailored to the individual’s needs [53].

Coordination of care and respect among team members also emerged as strong positive predictors of QoL improvements. Seamless coordination ensures that various aspects of patient care, such as medical management, rehabilitation, and psychosocial support, are delivered in a cohesive and complementary manner, minimizing the fragmentation and duplication of efforts [54]. Additionally, a culture of mutual respect within the team fosters an environment of trust, collaboration, and shared decision making, where each professional’s unique contributions are valued and integrated into the care plan [55].

Shared goal setting and patient involvement in care further reinforce the significance of patient-centered approaches within multidisciplinary teams. By actively engaging patients in setting their own health goals and incorporating their preferences and values into the care plan, a sense of empowerment and ownership is fostered, enhancing patient satisfaction and adherence to treatment [56,57,58,59]. This collaborative goal-setting process not only aligns the team’s efforts but also ensures that care interventions are tailored to the patient’s priorities and desired outcomes, ultimately contributing to improved QoL [60].

Interestingly, while problem-solving ability, access to shared patient records, and continuous professional development were significant predictors of QoL, their impact was slightly lower compared to the aforementioned factors. This suggests that while these elements are important contributors to effective multidisciplinary collaboration, the more fundamental aspects of communication, coordination, respect, and patient engagement may play a more pivotal role in driving positive QoL outcomes.

### 4.3. The Profound Impact of Social Support on Quality of Life

This study’s findings highlight the profound impact of social support on the QoL of older patients, underscoring the significance of addressing this aspect within the multidisciplinary care framework. Across all dimensions of social support examined—family, friendship, community, professional, and online networks—a consistent positive association with QoL was observed.

The strongest predictor of enhanced QoL was friendship support, reflecting the critical importance of peer relationships and social connectedness for older adults [61]. As individuals age, their social networks often shrink due to factors such as retirement, relocation, and the loss of loved ones [62]. Maintaining friendships and social ties can provide a sense of purpose, emotional support, and opportunities for engagement, all of which contribute to better mental and physical wellbeing [63].

Family support also emerged as a robust predictor of QoL, highlighting the foundational role of familial networks in the lives of older adults [64]. Family members often serve as primary caregivers, providing instrumental and emotional support, and acting as advocates for their loved ones’ needs [65]. By integrating family members into the multidisciplinary care team, their perspectives and caregiving roles can be better understood, and appropriate support services, such as respite care and caregiver education, can be provided to alleviate caregiver burden and enhance overall family wellbeing [66].

### 4.4. Social Support Variables

Social support plays a dual role in enhancing the effectiveness of multidisciplinary care and improving the overall quality of life (QoL) for elderly patients. It acts as a critical facilitator of integrated care by bridging communication between healthcare teams and patients, ensuring that care plans are not only well coordinated but also aligned with the patients’ preferences and needs [67]. Family members and close friends often help to articulate patient concerns, assist with medical appointments, and provide emotional support, thereby enhancing adherence to treatment plans and fostering a holistic care environment [68]. Additionally, robust social networks contribute directly to better QoL by offering emotional comfort, practical assistance, and a sense of belonging, which collectively bolster mental health, reduce feelings of isolation, and promote active engagement in health maintenance behaviors [69]. This interconnected support system effectively complements the multidisciplinary approach, resulting in more personalized, responsive care and improved health outcomes for older adults.

### 4.5. Theoretical Perspectives on the Impact of Multidisciplinary Collaboration on Quality of Life

This study highlights the significant positive impact of multidisciplinary collaboration on the quality of life (QoL) among older adults receiving primary care, as interpreted through several theoretical frameworks. The Socioemotional Selectivity Theory, which posits that older adults focus on emotionally meaningful goals and relationships, aligns with our finding that those aged 60–69 benefit most from multidisciplinary care (β = 0.32, *p* < 0.001). This suggests that early intervention through collaborative care resonates with their prioritization of positive social interactions and emotional wellbeing [70]. Furthermore, the Biopsychosocial Model supports the integration of biological, psychological, and social factors in enhancing QoL [71]. Our results indicate that factors such as gender, marital status, and the presence of chronic conditions interact with multidisciplinary care to produce significant improvements in QoL, demonstrating that holistic care addressing these interconnected domains is crucial for this population.

Additionally, the Social Support Theory underpins the observed benefits of multidisciplinary care in improving QoL by enhancing social networks and providing comprehensive support [72]. The Lubben Social Network Scale-6 (LSNS-6) findings suggest that participants with stronger social networks experience better outcomes, underscoring the value of support from healthcare professionals and caregivers in mitigating stress and isolation. The Social Cognitive Theory further explains the significant impacts of employment status and income level on QoL improvements, as higher income and employment provide better access to resources and social interactions, facilitating greater engagement with care [73]. The Life Course Perspective contextualizes these findings by considering how varying life experiences and health statuses influence the benefits of collaborative care across different age groups and conditions. Collectively, these theories provide a multidimensional understanding of why multidisciplinary collaboration effectively enhances QoL for older adults, emphasizing the need for tailored, integrative care approaches that address the diverse and evolving needs of this population.

### 4.6. Implications for Practice and Future Research

The findings of this study have significant implications for the design and implementation of primary care services for older adults. The clear benefits of multidisciplinary collaboration on QoL underscore the need for healthcare systems to prioritize and invest in the development of collaborative care models. This may involve restructuring care delivery processes, fostering interdisciplinary education and training, and establishing mechanisms for effective communication and coordination among diverse healthcare professionals. 

Furthermore, the identification of key drivers of effective multidisciplinary collaboration, such as effective communication, coordination, respect, and patient engagement, can inform the design of comprehensive training programs and protocols for healthcare teams. By promoting these critical elements, healthcare organizations can optimize the functioning of their collaborative care models and maximize the potential for positive QoL outcomes among older patients.

The profound impact of social support on QoL highlights the need for multidisciplinary teams to actively incorporate strategies for enhancing social connectedness and addressing social isolation among older adults. This may involve partnering with community organizations, facilitating peer support groups, providing caregiver education and respite services, and leveraging digital technologies to foster online social networks.

Additionally, the demographic variations observed in the study underscore the importance of tailoring care approaches to the specific needs and circumstances of different subgroups within the older adult population. Healthcare organizations should strive to develop culturally sensitive and inclusive care models that account for factors such as age, gender, marital status, and the presence of chronic conditions. This may involve specialized training for healthcare professionals, targeted outreach and education efforts, and the integration of diverse perspectives and experiences into the care planning process.

Future research should build upon these findings by exploring additional factors that may influence the effectiveness of multidisciplinary collaboration and its impact on QoL. This could include examining the role of organizational culture, leadership styles, and incentive structures within healthcare settings, as well as investigating the potential moderating effects of socioeconomic status, cultural backgrounds, and geographical locations.

### 4.7. Limitations of the Study

Despite providing valuable insights into the impact of multidisciplinary collaboration on the quality of life (QoL) among older patients receiving primary care, this study has several limitations that should be acknowledged. Firstly, the cross-sectional design limits the ability to establish causality between multidisciplinary collaboration and improvements in QoL. Longitudinal studies would be necessary to confirm causative relationships and to observe changes over time. Additionally, the sample is restricted to older adults aged 60 years and above from a specific geographical region in Al-Ahsa, Saudi Arabia, which may limit the generalizability of the findings to other populations or regions with different demographic and healthcare characteristics.

Another limitation is the reliance on self-reported measures for QoL, which may introduce response bias. Participants’ perceptions and ability to accurately recall their experiences might influence their responses, potentially affecting the reliability of the data. Moreover, while the study includes key demographic and health-related control variables, there may still be unmeasured confounders such as genetic factors, lifestyle choices, and environmental influences that were not accounted for, possibly affecting the outcomes.

The study also did not include detailed assessments of the specific roles and interactions within multidisciplinary teams, which could provide deeper insights into which aspects of collaboration are most beneficial. Furthermore, the exclusion of participants with severe cognitive impairments or acute psychiatric conditions means that the findings may not fully represent the experiences of all older adults receiving primary care.

## 5. Conclusions

The findings of this cross-sectional study provide robust evidence for the significant positive impact of multidisciplinary collaboration on the quality of life (QoL) for older patients receiving primary care. By leveraging the collective expertise and coordinated efforts of healthcare professionals from diverse disciplines, collaborative care models have demonstrated their potential to enhance physical functioning, mental health, and overall health perceptions among the elderly population.

Multidisciplinary collaboration emerges as a powerful approach to addressing the multifaceted challenges faced by older adults, including the proportion of chronic conditions, mobility limitations, cognitive decline, and social isolation. Through effective communication, coordination, shared goal-setting, and patient involvement, these collaborative teams can develop comprehensive care plans tailored to each individual’s unique circumstances, ultimately improving their overall wellbeing and promoting independence.

The study also highlights the profound impact of social support on QoL, underscoring the importance of actively fostering and strengthening social connections for older adults. By integrating strategies to enhance family, friendship, community, and professional support networks, multidisciplinary teams can mitigate the detrimental effects of social isolation and empower patients to maintain a sense of belonging and purpose.

Interestingly, the benefits of multidisciplinary collaboration were found to vary across demographic subgroups, with younger age, female gender, married status, and the absence of chronic conditions being associated with greater improvements in QoL. These findings emphasize the need for tailored care approaches that account for the unique needs and circumstances of different subpopulations within the older adult community.

## Figures and Tables

**Table 1:** Demographic and baseline characteristics of participants.

Characteristic		Total Participants (N = 200)
Age (years)	60–69	76 (38%)
	70–79	87 (43.5%)
	80+	37 (18.5%)
Gender	Male	92 (46%)
	Female	108 (54%)
Marital Status	Married	123 (61.5%)
	Widowed	54 (27%)
	Divorced/Separated	15 (7.5%)
	Single (never married)	8 (4%)
Educational Level	No formal education	20 (10%)
	Primary school	56 (28%)
	Secondary school	62 (31%)
	Higher education	62 (31%)
Living Arrangement	Alone	40 (20%)
	With spouse	100 (50%)
	With children	45 (22.5%)
	Other (relatives, nursing home)	15 (7.5%)
Chronic Conditions (number of conditions)	0	18 (9%)
	1–2	82 (41%)
	3–4	75 (37.5%)
	5+	25 (12.5%)
Employment Status	Employed	40 (20%)
	Retired	120 (60%)
	Unemployed	40 (20%)
Income Level	Low	60 (30%)
	Medium	100 (50%)
	High	40 (20%)

**Table 2:** Descriptive statistics for QoL, multidisciplinary collaboration, and social support.

Variable		Mean Score	Standard Deviation (SD)	Range
Quality of Life (QoL)	Physical Functioning (SF-36)	62.3	12.5	30–90
	Mental Health (SF-36)	60.8	14.2	25–95
Multidisciplinary Collaboration	Communication	75.2	8.3	50–95
	Coordination	73.6	9.1	45–90
	Goal Congruence	74.1	7.5	55–95
	Problem-solving Efficiency	72.8	10.2	40–90
Social Support	Lubben Social Network Scale (LSNS)	32.5	6.7	10–50

**Table 3:** Multivariate regression analysis of multidisciplinary collaboration impact on QoL.

Outcome Variable (SF-36 Dimension)		β	Standard Error	*p*-Value	95% CI	R-Squared	Adjusted R-Squared	F-Statistic
Physical Functioning	Multidisciplinary Collaboration	2.35	0.58	<0.001	[1.21, 3.49]	0.42	0.40	15.67
	Social Support (LSNS)	1.89	0.45	<0.001	[1.01, 2.77]			
	Age	−0.52	0.11	<0.001	[−0.73, −0.31]			
	Number of Chronic Conditions	−1.77	0.39	<0.001	[−2.53, −1.01]			
	Employment Status	1.20	0.50	0.014	[0.21, 2.19]			
	Income Level	1.40	0.55	0.010	[0.32, 2.48]			
Mental Health	Multidisciplinary Collaboration	3.01	0.62	<0.001	[1.78, 4.24]	0.38	0.36	13.89
	Social Support (LSNS)	2.47	0.50	<0.001	[1.49, 3.45]			
	Age	−0.31	0.10	0.002	[−0.50, −0.12]			
	Number of Chronic Conditions	−1.29	0.41	0.001	[−2.09, −0.49]			
	Employment Status	1.50	0.54	0.005	[0.43, 2.57]			
	Income Level	1.70	0.59	0.004	[0.54, 2.86]			
General Health Perceptions	Multidisciplinary Collaboration	2.12	0.55	<0.001	[1.04, 3.20]	0.36	0.34	12.53
	Social Support (LSNS)	1.75	0.47	<0.001	[0.83, 2.67]			
	Age	−0.28	0.09	0.004	[−0.45, −0.11]			
	Number of Chronic Conditions	−1.03	0.38	0.005	[−1.76, −0.30]			
	Employment Status	1.10	0.53	0.032	[0.06, 2.14]			
	Income Level	1.30	0.57	0.020	[0.18, 2.42]			

**Table 4:** Key factors within multidisciplinary teams contributing to quality of life in older patients.

Factor	β Coefficient	Standard Error	*p*-Value	95% Confidence Interval
Effective Communication	0.48	0.10	<0.001	0.28 to 0.68
Coordination of Care	0.42	0.09	<0.001	0.24 to 0.60
Shared Goal Setting	0.37	0.11	0.001	0.15 to 0.59
Problem-Solving Ability	0.31	0.12	0.010	0.07 to 0.55
Respect among Team Members	0.45	0.10	<0.001	0.25 to 0.65
Patient Involvement in Care	0.40	0.11	<0.001	0.18 to 0.62
Access to Shared Patient Records	0.35	0.13	0.005	0.09 to 0.61
Continuous Professional Development	0.25	0.10	0.020	0.05 to 0.45

**Table 5:** Impact of social support on QoL in older patients.

Social Support Dimension	β Coefficient	Standard Error	*p*-Value	95% CI
Family Support	0.28	0.06	<0.001	0.16 to 0.40
Friendship Support	0.33	0.07	<0.001	0.19 to 0.47
Community Support	0.21	0.07	0.003	0.07 to 0.35
Professional Support (PHC)	0.25	0.08	0.001	0.09 to 0.41
Online Social Networks	0.18	0.07	0.012	0.04 to 0.32

**Table 6:** Demographic variations in the impact of multidisciplinary collaboration on QoL.

Demographic Factor		N	β	Standard Error	*p*-Value	95% CI	R-Squared	Adjusted R-Squared	F
Age Group	60–69	76	0.32	0.07	<0.001	0.18 to 0.46	0.45	0.43	12.53
	70–79	87	0.27	0.08	0.001	0.11 to 0.43	0.42	0.40	11.67
	80+	37	0.15	0.10	0.137	−0.05 to 0.35	0.32	0.28	5.43
Gender	Male	92	0.25	0.07	0.002	0.11 to 0.39	0.40	0.38	10.25
	Female	108	0.30	0.06	<0.001	0.18 to 0.42	0.47	0.45	14.38
Marital Status	Married	123	0.29	0.06	<0.001	0.17 to 0.41	0.46	0.44	13.94
	Widowed	54	0.26	0.09	0.004	0.08 to 0.44	0.41	0.38	9.87
	Divorced/Separated	15	0.22	0.14	0.134	−0.06 to 0.50	0.31	0.26	4.76
	Single(never married)	8	0.31	0.21	0.156	−0.11 to 0.73	0.34	0.28	5.21
Chronic Conditions	0	18	0.40	0.12	<0.001	0.16 to 0.64	0.50	0.47	15.24
	1–2	82	0.30	0.06	<0.001	0.18 to 0.42	0.46	0.44	13.94
	3–4	75	0.22	0.07	0.003	0.08 to 0.36	0.40	0.37	10.32
	5+	25	0.18	0.09	0.037	0.00 to 0.36	0.37	0.33	8.21
Employment Status	Employed	40	0.27	0.08	0.001	0.11 to 0.43	0.43	0.40	11.76
	Retired	120	0.29	0.07	<0.001	0.15 to 0.43	0.46	0.44	13.58
	Unemployed	40	0.24	0.09	0.005	0.06 to 0.42	0.41	0.38	10.65
Income Level	Low	60	0.25	0.08	0.002	0.09 to 0.41	0.42	0.40	11.48
	Medium	100	0.31	0.07	<0.001	0.17 to 0.45	0.48	0.46	14.87
	High	40	0.28	0.09	0.003	0.10 to 0.46	0.44	0.41	12.35

## Data Availability

Data are available upon request.
